# Classification of Data from Electronic Nose Using Gradient Tree Boosting Algorithm

**DOI:** 10.3390/s17102376

**Published:** 2017-10-18

**Authors:** Yuan Luo, Wenbin Ye, Xiaojin Zhao, Xiaofang Pan, Yuan Cao

**Affiliations:** 1 School of Electronic Science and Technology, Shenzhen University, Shenzhen 518060, China; tongxueluo@gmail.com (L.Y.); eexjzhao@szu.edu.cn (X.Z.); caoyuan0908@gmail.com (C.Y.); 2 Key Laboratory of Optoelectronic Devices and Systems of Ministry of Education and Guangdong Province, College of Optoelectronic Engineering, Shenzhen University, Shenzhen 518060, China; 3 School of Information Engineering, Shenzhen University, Shenzhen 518060, China; eexpan@szu.edu.cn

**Keywords:** electronic nose, gas sensors, gradient tree boosting, fast recognition

## Abstract

In this paper, an approach that can fast classify the data from the electronic nose is presented. In this approach the gradient tree boosting algorithm is used to classify the gas data and the experiment results show that the proposed gradient tree boosting algorithm achieved high performance on this classification problem, outperforming other algorithms as comparison. In addition, electronic nose we used only requires a few seconds of data after the gas reaction begins. Therefore, the proposed approach can realize a fast recognition of gas, as it does not need to wait for the gas reaction to reach steady state.

## 1. Introduction

An electronic nose, which imitates the perceptional mechanisms of biological olfactory organ, has been widely used in many applications such as the medical and diagnostic [1,2], food [3,4,5] and environment [6,7,8,9,10,11] monitor.

One important part of an electronic nose system is a pattern recognition system that would recognize the olfactory of the tested gas. Therefore, in the past decades, many pattern recognition algorithms have been introduced for the gas classification. In [12,13,14], a simple but quite effective method, the *K*-nearest neighbor (KNN) was first introduced in electronic nose applications for gas classification. The Gaussian mixture model (GMM) method [15,16] is also explored for the gas classification. Though Both KNN and GMM methods are simple, they suffer a limitation that their accuracy is limited when the size of train data is small. A binary decision tree (BDT) is first proposed in [17]. The BDT is easy understand and friendly to hardware implementation, but it is unstable and its accuracy is not high. In order to cope with nonlinearity of gas classification problem and to improve the classification accuracy, the advanced methods such as artificial neural networks (ANN) like multiple layer perception (MLP) [18,19,20], restricted boltzmann machines (RBM) [21,22], support vector machine (SVM) [23,24,25] and relevance vector machine(RVM) [26,27] are also presented. Despite the fact that these advanced methods [18,19,20,21,22,23,24,25,26,27] could provide the a high accuracy classification, a significant and practical disadvantage of these methods is that they can not directly handle the raw, time-sampled sensor response data due to the high dimensional patterns. In other words, a preprocessing block that extract the features from the raw data is necessary for the above mentioned advanced methods. Since featuring extracting is not straightforward and generally needs very complexity processing techniques, which will lead to the significantly increase of the power consumption and system complexity.

In order to overcome the limitations either on low accuracy or needing to extract features from raw data, in this paper, gradient tree boosting algorithm which could direct handle the raw, time-sampled sensor data is first introduced to the gas classification. Compared with conventional methods, the proposed methods have the following advantages [28]: (1) It can handle high-dimensional features without additional feature engineering; (2) Robust to overfitting; (3) Can naturally deal with the nonlinearity in the classification; (4) can provide high classification accuracy even with small size of train data.

Besides that, the proposed algorithm can realize the fast classification with high accuracy. Though there many techniques have been proposed to extract transient features [29,30,31,32] by performing certain operation on raw sampled data such as doing the exponential moving average or derivative to realize fast classification, no one have proposed to use the raw sampled data as transient features.

The rest of this article is organized as follows. In the next section, the proposed gradient tree boosting algorithm is presented. Section 3 discusses the experimental results that compare the performance of the classification accuracy of different classifier methods. Some concluding remarks are given in Section 4.

## 2. Gradient Tree Boosting Algorithm

Machine-learning techniques have been becoming more and more prevalent in many areas. Among the machine-learning algorithms, gradient tree boosting has shown huge success in many applications. On classification benchmarks gradient tree boosting achieved the leading results [33], ranging from ranking problems to rate prediction problem [34]. Since its invention [35], the recent development further advanced the advantage of the tree boosting algorithm. The Extreme Gradient Boosting, or called XGBoost [36], is a scalable tree boosting system. Due to several important optimizations in split finding and system design, XGBoost has achieved great success and been prevalently used in the winning teams in major data competitions like Kaggle and Knowledge Discovery and Data Mining cup (KDDCup) [36]. In the following of this chapter, the gradient tree boosting algorithm tailored for the gas classification is discussed.

### 2.1. Tree Ensemble and Learning Objective

**Figure 1:**
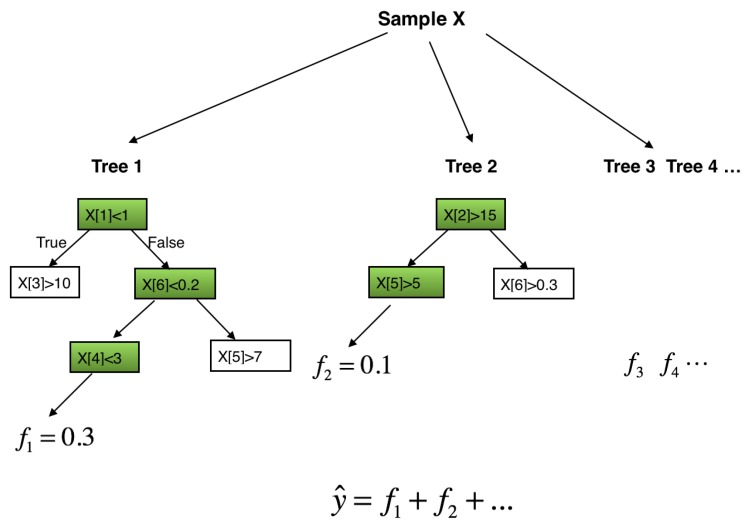
Tree Ensemble Model. The final prediction $\hat{y}$ for an instance $x_{i}$ is the sum of predictions from each tree.

Considering the given data set as $D=\{\left(x_{i},y_{i}\right)\}$, with $x_{i}$ representing the feature for data instance *i* (assuming $x_{i}\in R$) and $y_{i}$ its target. Assume the number of data instances is *n* and the dimension of feature vector is *m*. For a tree ensemble model, the output ${\hat{y}}_{i}$ is predicted by summing *K* additive functions:

(1) $${\hat{y}}_{i}=\phi \left(x_{i}\right)=\sum_{k=1}^{K}f_{k}\left(x_{i}\right),$$

where $f_{k}\left(x_{i}\right)$ is the prediction given by the *k*-th classification and regression tree (CART) [37]. Figure 1 depicts the ensemble tree model.

Denote the number of leaves in a single CART as *T*, and define the structure of the tree that maps the data instance *x* to the corresponding leaf index as $q\left(x\right):R^{m}\to T$. Then in Equation (1), the prediction $f_{k}\left(x_{i}\right)$ of *k*-th CART for *i*-th data instance $x_{i}$ can be written as

(2) $$f\left(x_{i}\right)=w_{q(x_{i})}$$

where *w* denote the leaf weights of the CART and $q(x_{i})$ represents the mapping function defined by the tree structure.

Then, the learning objective of the tree ensemble model can be set to minimize the following loss function:

(3) $$L\left(\phi \right)=\sum_{i=1}^{n}l\left({\hat{y}}_{i},y_{i}\right)+\sum_{k=1}^{K}\Omega \left(f_{k}\right),$$

where the differentiable convex loss function $l({\hat{y}}_{i},y_{i})$ measures the difference the target $y_{i}$ and the prediction ${\hat{y}}_{i}$ in Equation (1), and in the summation, *n* represents the number of data instances, *K* is the number of CARTs used in the algorithm. Here Ω(f) are the regularization terms for penalizing the model’s complexity and avoiding overfitting, defined by the number of leaves *T* and the square of leaf weights *w*:

(4) $$\Omega \left(f\right)=\gamma T+\frac{1}{2}{\lambda ||w||}^{2},$$

where γ and λ are regularization parameters.

### 2.2. Gradient Boosting Algorithm

Here the goal of training the model is to minimize the overall loss function L(ϕ). However, traditional optimization methods cannot apply to minimize it in Euclidean space, since the loss function of the tree ensemble model in Equation (3) depends on each tree’s structure as well as parameters. To solve this problem and efficiently achieve this goal, the gradient tree boosting algorithm is proposed and developed [35,36,38]. In the following let us review the algorithm.

In training the model, at the (t−1)-th iteration, define the loss function as

(5) $$L^{\left(t-1\right)}=\sum_{i=1}^{n}l\left(y_{i},{\hat{y}}_{i}^{\left(t-1\right)}\right)+\sum_{k=1}^{t-1}\Omega \left(f_{k}\right)$$

where ${\hat{y}}_{i}^{\left(t-1\right)}$ represents the prediction of the *i*th instance at the (t−1)th iteration. Then, to minimize it we additively add $f_{t}$ at *t*-th iteration, and the loss function becomes

(6) $$L^{\left(t\right)}=\sum_{i=1}^{n}l\left(y_{i},{\hat{y}}_{i}^{\left(t-1\right)}+f_{t}\left(x_{i}\right)\right)+\sum_{k=1}^{t-1}\Omega \left(f_{k}\right)+\Omega \left(f_{t}\right).$$

In other words, we greedily add the tree which can most improves the model according to Equation (3). Therefore, at iteration *t* where we add the *t*-th CART, the objective is to find the tree structure of *t*-th CART that defines $f_{t}$ and $\Omega (f_{t})$, to minimize $L^{\left(t\right)}$.

Firstly, note that by Taylor expansion, we can write the loss function as

(7) $$\begin{array}{cc} L^{\left(t\right)}= & \sum_{i=1}^{n}\left[l\left(y_{i},{\hat{y}}^{\left(t-1\right)}\right)+g_{i}f_{t}\left(x_{i}\right)+\frac{1}{2}h_{i}f_{t}^{2}\left(x_{i}\right)\right]\\ & +\sum_{k=1}^{t-1}\Omega \left(f_{k}\right)+\Omega \left(f_{t}\right), \end{array}$$

with

(8) $$\begin{array}{cc} g_{i}= & \partial_{{\hat{y}}^{\left(t-1\right)}}l\left(y_{i},{\hat{y}}^{\left(t-1\right)}\right)\;\mathrm{and}\\ h_{i}= & \partial_{{\hat{y}}^{\left(t-1\right)}}^{2}l\left(y_{i},{\hat{y}}^{\left(t-1\right)}\right) \end{array}$$

representing the first and second order gradient statistics on $l(y_{i},{\hat{y}}^{\left(t-1\right)})$ respectively. At step *t*, the previous tree structures at t−1 are fixed and their loss function $\sum_{i=1}^{n}l\left(y_{i},{\hat{y}}^{\left(t-1\right)}\right)+\sum_{k=1}^{t-1}\Omega \left(f_{k}\right)$ can be seen as constant, thus we can remove it and obtain the simplified learning objective at step *t*

(9) $${\tilde{L}}^{\left(t\right)}=\sum_{i=1}^{n}\left[g_{i}f_{t}\left(x_{i}\right)+\frac{1}{2}h_{i}f_{t}^{2}\left(x_{i}\right)\right]+\Omega \left(f_{t}\right).$$

Then, using Equations (2) and (4), this equation leads to

(10) $${\tilde{L}}^{\left(t\right)}=\sum_{p=1}^{T}\left[\left(\sum_{i\in I_{p}}g_{i}\right)w_{p}+\frac{1}{2}\left(\sum_{i\in I_{p}}h_{i}+\lambda \right)w_{p}^{2}\right]+\gamma T.$$

Here $I_{p}=\left\{i|q\left(x_{i}\right)=p\right\}$ represents the data instance set of leaf *p*, i.e., all the instances that are mapped to leaf leaf *p*.

Then, for a fixed tree structure q(x), the optimal weight $w_{p}^{opt}$ of leaf *p* is defined by the minimization equation

(11) $$\partial_{w}{\tilde{L}}^{\left(t\right)}=0.$$

with ${\tilde{L}}^{\left(t\right)}$ defined by Equation (10), this function gives solution

(12) $$w_{p}^{opt}=-\frac{\sum_{i\in I_{p}}g_{i}}{\sum_{i\in I_{p}}h_{i}+\lambda }.$$

Taking this optimal weight $w_{p}^{opt}$, the corresponding optimal loss function of Equation (10) becomes

(13) $${\tilde{L}}_{opt}^{\left(t\right)}=-\frac{1}{2}\sum_{p=1}^{T}\frac{{\left(\sum_{i\in I_{p}}g_{i}\right)}^{2}}{\sum_{i\in I_{p}}h_{i}+\lambda }+\gamma T.$$

Therefore, for each iteration *t* of constructing the *t*-th CART, our goal becomes finding its best tree structure *q* that gives the minimum ${\tilde{L}}_{opt}^{\left(t\right)}$.

However, in finding the best *q*, enumerating all possible tree structures is not practical. Instead, the greedy algorithm is used. Starting from a single leaf, we can iteratively split the tree nodes and add branches to the tree. For each iteration, denote the data instance set as *I* before splitting, and denote $I_{L}$ and $I_{R}$ respectively to be the instance sets of the left and right nodes after splitting. Because $I=I_{L}\cup I_{R}$, using Equation (13), the loss function reduction after this splitting is given by

(14) $${\mathrm{LR}}_{split}=\frac{1}{2}\left[\frac{{\left(\sum_{i\in I_{L}}g_{i}\right)}^{2}}{\sum_{i\in I_{L}}h_{i}+\lambda }+\frac{{\left(\sum_{i\in I_{R}}g_{i}\right)}^{2}}{\sum_{i\in I_{R}}h_{i}+\lambda }-\frac{{\left(\sum_{i\in I}g_{i}\right)}^{2}}{\sum_{i\in I}h_{i}+\lambda }\right]-\gamma .$$

Therefore, to find the best tree structure, the algorithm iteratively adds the branches by choosing the splitting that maximizes ${\mathrm{LR}}_{split}$.

## 3. Experimental Setup and Performance Evaluation

### 3.1. Experimental Setup and the Measurement Procedure

**Figure 2:**
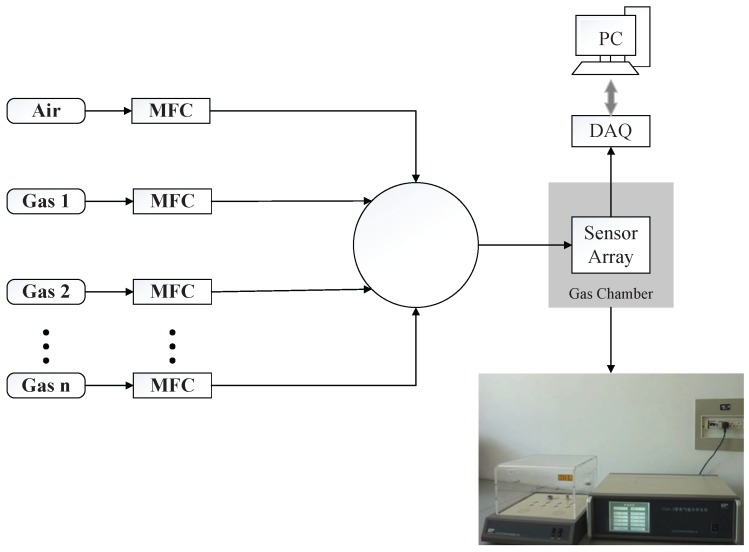
Experimental setup to acquire signatures of the target gases with the sensor array.

A block diagram of the automated gas delivery setup used to acquire the signatures of the target gases with the sensor array is shown in Figure 2. Eight commercial Figaro metal oxide semiconductor (MOS) sensors with diverse sensing performance are used to build the gas sensor array and their corresponding part numbers are listed in Table 1. As the working temperature of the sensor, which is controlled with a built-in heater and thus the voltage in sensor heater is also listed in Table 1. The electronic signal of these sensors are simultaneously acquired through chemical gas senor CGS-8 system in 10Hz sampling rate. Computer controlled mass flow controllers (MFCs) are used to control the flow rate of the target gas. Through changing the ratio of the flow rate between target gas and background gas, we can get a range of concentrations of target gas. For example, in the case that the background gas is air and the target gas is Methane $\left({\mathrm{CH}}_{4}\right)$. If we would like to set the target methane gas at concentration 100 ppm, we can first buy a bottle of methane with original concentration 500 ppm and then set the ratio of flow rate between air and methane to 4:1. Therefore, if we have a bottle of methane with concentration 500 ppm, any concentration between 0 to 500 ppm can be achieved by properly controlling the flow rate between air and methane through the MFCs. Before the gas reaction, the air is injected to the chamber for 500 s to clean the surface of gas sensors and get a stable baseline resistance. Then, thesensor array is exposed to the reaction gas for 160 s to ensure sensors reach the saturation status. In our case, six type gases, i.e., Carbon Monoxide (CO), methoxymethane $\left(C_{2}H_{6}O\right)$, Ethylene $\left(C_{2}H_{4}\right)$, Methane $\left({\mathrm{CH}}_{4}\right)$, Ethane $\left(C_{2}H_{6}\right)$ and Hydrogen $\left(H_{2}\right)$ are used for the reaction. The concentration ranges for each target gas is from 20 ppm to 200 ppm with a stepsize 20 ppm. The reason why we chose these 6 gases is that they are the most common inflammable and explosive gases which may result in great damage when they leaked. The low and upper explosive levels for these gases are 12–74.2% VOL, 3.3–19% VOL, 2.7–36% VOL, 5–15% VOL, 3–12.4% VOL and 4.1–74.2% VOL, where 1% V is 10,000 ppm. And we think that realization the fast recognition of these 6 gases may help to prevent a conflagration in Petrochemical industry or in daily life.

### 3.2. Data Set and features

**Figure 3:**
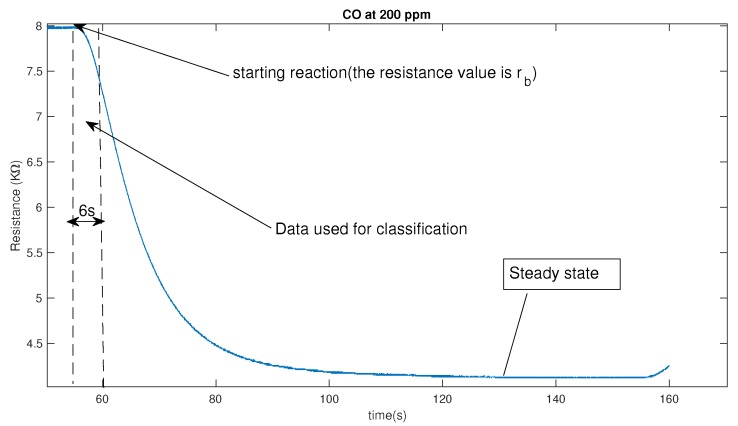
The response of a metal-oxide based chemical sensor to 200 ppm of CO.

As there are 10 concentrations for each gas, for each type gas at each concentration, we make 25 repeated measurements and thus there are 250 measurements for each type gas. As there are 6 types in our case, totally we have 250×6=1500 samples in our data sets. It should be noted that the proposed algorithm can be directly applied to the raw data set and thus there is no need to do the preprocessing of the sampled raw data for the feature extraction. And, it is one advantage of the proposed algorithm, which can directly handle high-dimensional data without any feature extraction engineering. Besides that, we found that it only a small part of the time-sampled raw data is sufficient for the high-accuracy classification. In our case, we only use the first 6 s raw data since the reaction of the sensor started as shown in Figure 3. From the Figure 3 , at 200 ppm for gas CO, the gas sensor TGS2602 takes about 75 s to reach the steady state. Therefore, compared with existing method which use the sensor resistance at steady state as important features for the recognition, the proposed method can realize the recognition 12 time faster.

The first 6 s raw data (resistance value) of each sensor is directly used as features. As the sample rate of data acquisition device (DAQ) is 10 Hz, i.e., there are 6×10=60 features of for *i*-th sensor at *j*-th measurement, which can be denoted as $R_{j}^{i}=\left[r_{j1}^{i},r_{j2}^{i},r_{j3}^{i},\ldots ,r_{j60}^{i}\right]$, where *r* represents the resistance value at certain time. In our E-nose, there are 8 gas sensors and thus there are 8×10×6=480 features in total for *j*-th measurement, which can be denoted as $F_{j}=\left[R_{j}^{1},R_{j}^{2},R_{j}^{3},\ldots ,Rj^{8}\right]$. In order to reduce impact of the baseline drift, the final features vector the baseline resistance, denoted as $r_{b}$, which is the resistance of gas sensor before starting reaction is subtracted from feature vector, i.e., the final feature vector can be expressed as $F_{j}-r_{b}$.

### 3.3. Results

For each type gas, the dataset consisted of 250 samples is randomly split into 70% training and 30% test sets. We used the same training gas sets to train the different classification algorithms, including the proposed one. And the test set is also the same between the proposed algorithm and the comparison algorithms. In other words, all the algorithms are learned from same data and test their classification accuracy on the same data. Therefore, under this circumstance, the algorithm with highest classification accuracy should be best one. Classification accuracy is one of the most important evaluation metrics for supervised learning algorithms and it can be obtained by the number of correctly recognized examples is divided by the total number of testing examples. Moreover, in order to do a fair comparison with the GMM, KNN, MLP, and SVM methods, the same condition (the same raw sampled data) is also applied to these methods, though generally some preprocessing techniques such as Principal Component Analysis (PCA), fast fourier transform (FFT) and discrete wavelet transform (DWT) should be applied to raw data to extract features when the GMM, KNN, MLP, and SVM methods are used. In addition, as the training set and test set are randomly selected from the whole dataset, to eliminate the bias of the test result, we repeated this train-test procedure 100 times with different random splits. Then we average the accuracy of each test to get the accuracy for each classifier.

Table 2 shows the classification performance for various algorithms. It can be seen from Table 2, the GMM reach the lowest accuracy. In the GMM model, it assumes that the probability distribution of observations in the overall population can be represented by mixture Gaussian distribution, but this assumption is not always satisfied. Moreover, estimating the covariance matrices for the Gaussian components becomes difficult when the feature space gets large and is comparable to the number of the data points. Therefore, in our case where the dimension of feature space can get as large as 480, the classification performance of GMM is significantly poor. Table 2 also shows that the proposed gradient tree boosting achieves the highest classification accuracy. It verifies the claim that the proposed gradient tree boost algorithm can handle high-dimensional features without additional feature engineering and still achieve high accuracy while the existing methods such as GMM, KNN and SVM can not. Without any additional feature engineering, the raw sampled data can be directed taken as the input of the proposed gradient tree boost method, which could lead to the fast recognition of the gas.

**Figure 4:**
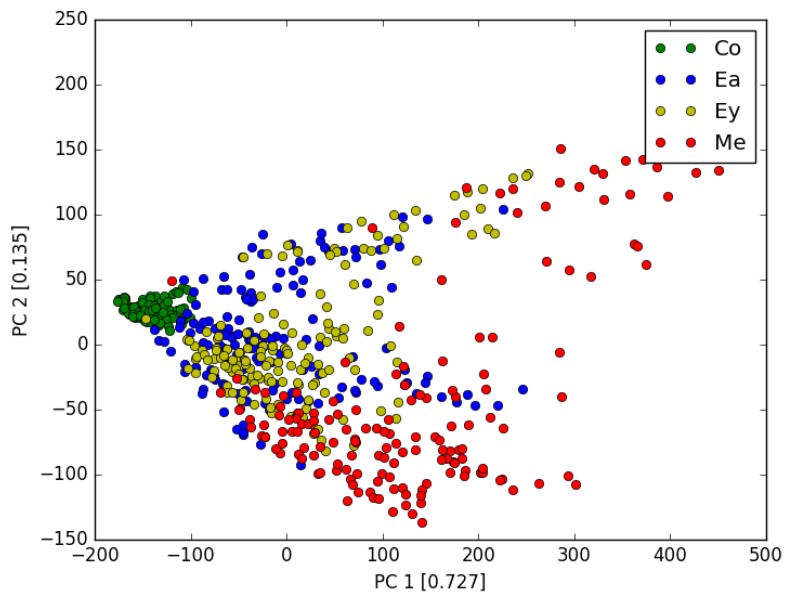
2-dimensional PCA plot of 4 gases.

Here to make the analysis more complete, we also tested the following approach. We first use the PCA to reduce the dimension of feature space from 360 to 10, with the total explain variance ratio >0.995. Then test each algorithm with the PCA features. The performances are listed in Table 3. We found that for each algorithm, PCA processing does not improve the accuracy (We also tested other different numbers of PCA components, the results are similar.). The Figure 4 shows the first 2 most dominant components (with explain variance ratio of 0.727 and 0.135 respectively) for 4 gases. The reason why PCA cannot significantly improve the accuracy can be seen from Figure 4 . We can see that different gases have different properties, but the boundary of each gas is not clear define. In other words, for the existing algorithm such as KNN, GMM, MLP and SVM, more sophisticated feature extracting engineering is required to further improve the accuracy. Moreover, though the PCA processing reduces the feature dimension, it eliminates certain useful information when removing the noise.

### 3.4. An Example of Application Based on Raw Data to Realize Fast Recognition

Although natural gas (mainly consist of Methane ${\mathrm{CH}}_{4}$) is environmental friendly, it can lead to a serious damage if they leak. It is stored in pressurized steel cylinders in liquid form and vaporize at normal temperatures. When it leaks and reach certain concentration, ignition may happen and cause an explosion. Therefore, the detection of Methane leakage as early as possible is quite desirable. In order to test whether the proposed algorithm based on raw sampled data can realize the fast detection the Methane leakage on real application in a open environment, we lift the glass cover of the gas chamber so the sensor array can exposed to real environment. Then, we turn on the valve of methane bottle and let methane gas leak through a rubber pipe which is placed near the sensor array. The leakage only last for 6 s and the 6 s raw sampled data collected by the sensor system is direct fed into the proposed gradient tree boost classifier which will recognize whether the methane is existed or not. Such measurements are repeated for 40 times, and the proposed gradient tree boost classifier recognizes the sample correctly as methane 39 times, while GMM, KNN, MLP and SVM classifiers only reach 13, 31, 28, 34 times. In other words, even in an open environment, the proposed gradient tree boost classifier can realize fast detection of methane leakage with high accuracy, which may help to prevent a fire cased by natural gas leakage.

## 4. Conclusions

In this paper, we applied Gradient tree boosting algorithm to solve the multi-classification problems for 6 different gases. We showed that this algorithm achieved higher performance than that of conventional algorithms. Besides, since the approach we used only need to take the first few seconds data of the electronic nose after gas reaction, without any additional feature engineering, it is able to detect certain gas quickly and efficiently. Therefore, our approach could have great potential in practical application.

## Figures and Tables

**Table 1:** Types of metal oxide semiconductor (MOS) sensors (provided by Figaro Inc.)

Channel	Sensor Part Number	Voltage in Sensor Heater
0	TGS821	5 V
1	TGS812	5 V
2	TGS2610	5 V
3	TGS2612	5 V
4	TGS3870	5 V
5	TGS2611	5 V
6	TGS816	5 V
7	TGS2602	5 V

**Table 2:** Classification Performance of Algorithms.

Classifier	Accuracy(%)
GMM [15]	25.7
KNN [12]	84.6
MLP [20]	86.9
SVM [24]	86.2
The Proposed Gradient Tree Boosting Algorithm	96.9

**Table 3:** Classification Performance of Algorithms after PCA Preprcessing.

Classifier	Accuracy (%)
GMM [15]	25.9
KNN [12]	84.5
MLP [20]	86.7
SVM [24]	86.3
The Proposed Gradient Tree Boosting Algorithm	96.7
